# Periprosthetic patella fractures in total knee replacement and revision surgeries: how to diagnose and treat this rare but potentially devastating complication—a review of the current literature

**DOI:** 10.1007/s00590-023-03535-9

**Published:** 2023-03-31

**Authors:** Virginia Masoni, Fortunato Giustra, Francesco Bosco, Lorenzo Lo Carmine, Marcello Capella, Giorgio Cacciola, Salvatore Risitano, Luigi Sabatini, Alessandro Massè

**Affiliations:** 1 Department of Orthopaedics and Traumatology, University of Turin, CTO, Via Zuretti 29, 10126 Turin, Italy; 2 Department of Orthopaedics and Traumatology, Ospedale San Giovanni Bosco di Torino - ASL Città di Torino, Turin, Italy; 3 Istituto Ortopedico del Mezzogiorno d’Italia “Franco Scalabrino”, Via Consolare Pompea, 98100 Messina, Italy

**Keywords:** Periprosthetic patellar fractures, Periprosthetic, Resurfaced, Un-resurfaced, Patella, Total knee arthroplasty, TKA, Total knee replacement, TKR, Extensor apparatus, Failure, Prevention, Review

## Abstract

**Purpose:**

Periprosthetic patella fractures (PPPFs) are infrequent but potentially devastating complications after total knee arthroplasty (TKA) and revision TKA (rTKA). These fractures may occur both in resurfaced and un-resurfaced patella. This review summarizes the current literature on PPPFs to help orthopedic surgeons diagnose and treat this uncommon but extremely challenging TKA complication.

**Methods:**

A comprehensive search was performed in three databases: PubMed, SCOPUS, and EMBASE. All relevant information was retrieved and summarized in this narrative review.

**Results:**

In the studies analyzed, there is a general trend in favor of nonsurgical treatment, except for implant loosening or extensor lag with extensor apparatus disruption, because surgery is often associated with poor clinical outcomes and high complication rates.

**Conclusion:**

PPPF is a rare but catastrophic event in TKA and rTKA, occurring mainly in a reconstructed patella. Patient-, implant-, and surgical technique-related factors contribute to its multifactorial etiopathogenesis. Prevention plays a crucial role in reducing the PPPFs rate. Conservative management is the treatment of choice due to high surgery complication rates unless implant loosening, or extensor apparatus disruption occurs.

## Introduction

Periprosthetic patella fractures (PPPF) are a challenging complication in total knee arthroplasty (TKA) and revision TKA (rTKA). In the literature, PPPFs are reported on average in 1.19% of TKAs; in 99% of cases, these occur in patients undergoing patellar replacement [1–5]. Currently, no PPPFs classification system is universally validated; the most widely used are those of Goldberg et al. [6] and Ortiguera and Berry [1]. The pathogenesis is multifactorial, and many different predisposing risk factors are reported in the literature [3, 4, 7–9]. Most fractures are not associated with direct trauma but are discovered during follow-up [4]. Several treatment algorithms are described, but there has yet to be a universal consensus about the optimal management of these fractures [1, 4, 6–10]. A conservative approach is usually reserved for nondisplaced, stable implant fractures; surgical procedures are planned for patellar implant mobilization or extensor apparatus disruption with poor outcomes and high complication rates [1–4, 6–9]. This study aims to analyze the current literature on PPPFs, focusing on predisposing risk factors and preventive strategies to support the orthopedic surgeon in properly managing this rare but devastating complication in TKAs.

### Search strategy

A comprehensive narrative review of the current literature on PPPFs examining epidemiology, risk factors, clinical manifestations, classification systems, diagnosis, management, outcomes, and preventive strategies was performed. The search was conducted using the PubMed, EMBASE, and SCOPUS databases.

### Epidemiology

**Fig. 1:**
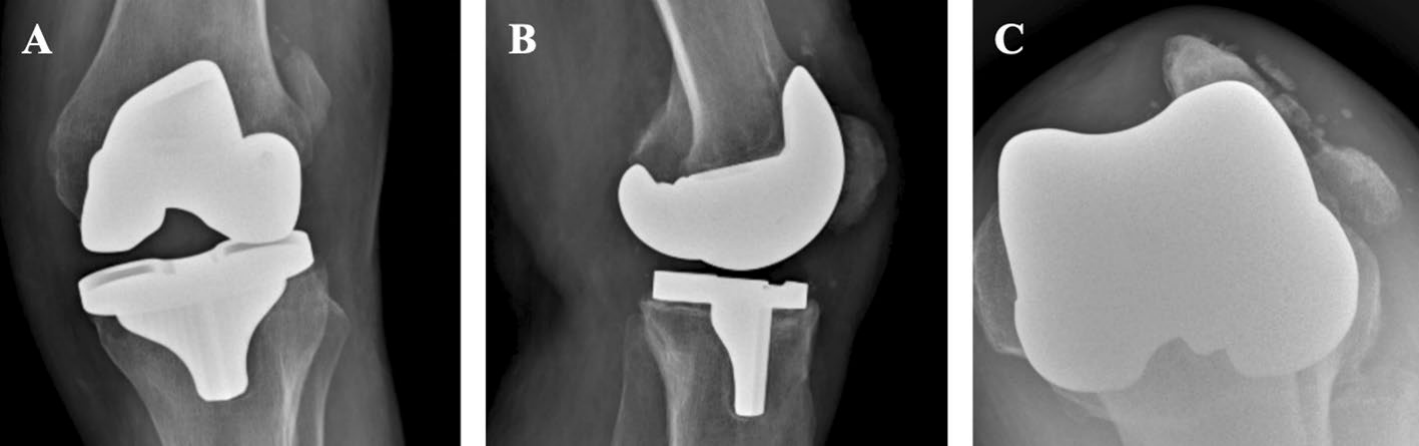
Periprosthetic patellar fracture in the non-resurfaced setting with failure of the extensor apparatus in a left knee. **A** Anteroposterior X-ray view; **B** Lateral X-ray view; **C** Merchant X-ray view

**Fig. 2:**
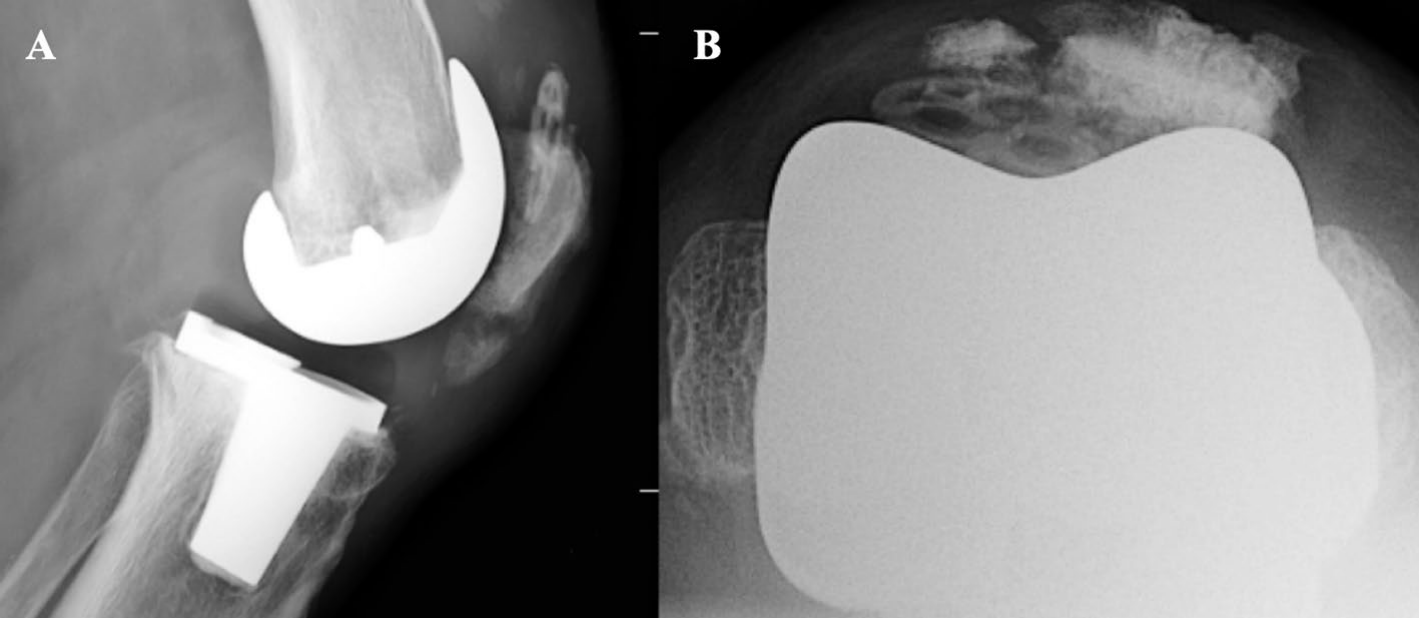
Periprosthetic patellar fracture in the resurfaced setting with loosening of the patellar button but an intact extensor apparatus. **A** Lateral X-ray view; **B** Merchant X-ray view

The PPPFs are a patellar complication in TKA [11] and represent the second most common periprosthetic fracture around the knee, after supracondylar femur fractures [12]. The PPPFs may occur in both resurfaced and non-resurfaced patella. Resurfacing is one of the most important predisposing factors, with a PPPFs prevalence of about 0.2–21% higher in the resurfaced patella than in the non-resurfaced one (Fig. 1) [3, 4, 8, 9]. Chalidis et al. [4] in their systematic review, reported a rate of 0.9% (5 cases) of fractures that occurred in a non-resurfaced patella, while the remaining 577 cases (99.1%) occurred in a patella resurfaced (Fig. 2).

PPPFs may occur intraoperatively, but in most cases, they are observed in the postoperative follow-up with an incidence that increases sharply during rTKA [3–5, 13]. In the Mayo Clinic joint registry, Berry reported that PPPFs are more frequent in rTKA than in primary TKA [5]. Furthermore, PPPFs usually occur in the first few years after TKA [1–4]. Chalidis et al. [4] described a mean time from surgery to fracture occurrence of 18.5 months.

Several studies recorded a male prevalence and identified the male sex as an independent risk factor [1, 3, 8]. Ortiguera and Berry [1] reported a prevalence of 1.01% and 0.40% in men and women, respectively, consistent with Parvizi et al. [3] series, where PPPFs occurred with a 2:1 ratio of males to females. These data differ from other periprosthetic knee fractures, which frequently occur in osteoporotic women [1, 8, 12]. The reason is unclear, but a possible cause is that men usually have a higher body weight and activity level, leading to greater forces damaging the extensor apparatus [1, 3, 8, 9]. Contrary to previous papers, other authors [6, 14] have reported a female prevalence, explained by a higher osteoporosis rate in women.

### Risk factors

The PPPFs may be traumatic or not [1–4, 8–10]. Although the literature seems to be a trend to atraumatic causes, there is much heterogeneity [1–4, 8–10, 14]. Chalidis et al. [4] described only 11.7% of cases associated with a traumatic event, whereas the recent case series by Govil et al. [14] reported that 66% of PPPFs were trauma related. The traumatic fracture may occur due to direct trauma, such as a fall on the knee, or indirect one, for example, an eccentric muscle contraction [1, 9, 15]. Ortiguera and Berry described several indirect mechanisms, like standing from a deep-seated position, hyperflexion, and knee overload [1]. Excluding traumatic cases, the literature strongly accords that the PPPF etiology is multifactorial [3, 4, 8, 9]. Nontraumatic PPPFs, therefore, are much more common than traumatic PPPFs, and most of them are related to fatigue fractures due to thinning of the native bone by loosening the patellar component or excessive original resection resulting from failure with the surgical technique. Several authors [3, 4, 8, 9] classified risk factors into three main groups: patient-related, implant-related, and surgical technique-related risk factors (Table 1).

**Table 1:** Summary of risk factors for atraumatic periprosthetic patellar fractures following TKA

Patient-related risk factors	Implant-related risk factors	Surgical technique-related risk factors
• Osteoporosis and osteopenia [3, 4]	• Resurfaced patella [3, 4, 8, 9]	• Patellar devascularization [4, 8, 9]
• Obesity [3, 4]	• Single central peg [3, 8, 9]	- Excessive fat pad removal
• Male sex [3, 8]	• Press-fit, cementless implant [3, 4, 8, 9]	- Excessive lateral meniscal removal
• Rheumatoid arthritis [8, 9]	• Inset design [3, 4]	- Lateral release
• High activity level [3, 4, 8]	• PMMA necrosis [3, 4, 8, 9]	- MA
• Poor bone stock [3, 4, 8, 9]	• PS TKA [3, 4, 8, 9]	• Excessive resection [3, 4, 8, 9]
• Bone cysts [3, 4, 8, 9]	• Metal-backed prosthesis [3, 4, 8, 9]	• Unsuitable thickness [8, 9]
• Excessive range of motion [3, 4, 9]		• Component mispositioning [3, 4, 8, 9]
• Previous surgery (revision) [3, 4, 8, 9]		- Femoral component in excessive flexion
		• Large AP diameter of femoral component [3, 4, 8, 9]
		• Patellar maltracking [9, 10]
		• Limb malalignment [8–10]

*PMMA* poly-methyl-methacrylate, *MA* medial arthrotomy, *PS* posterior-stabilized, *TKA* total knee arthroplasty, *AP* anteroposterior

#### Patient-related risk factors

Male sex seems to be a predisposing factor, whereas, regarding age, there is no definite tendency [3, 4, 8]. It is established that elderly patients have a higher risk of falls and osteoporosis [12]; Govil et al. [14] reported older age as a risk factor for PPPF, while Parvizi et al. [3] underlined that a young, active patient usually has a high degree of activity predisposing to a traumatic event. Moreover, a high activity level with knee hyperflexion is associated with a greater PPPF risk [3, 4, 9]. Osteoporosis, osteolysis, bone loss, and bone cysts are all associated with an increased PPPF risk. Lastly, rheumatoid arthritis (RA) is an independent predisposing factor because affected patients usually have poor bone quality due to the disease and the steroid medications assumed [3, 4, 8, 9].

#### Implant-related risk factors

The most common implant-related PPPF predisposing factor is patellar resurfacing [3, 4, 8]. Currently, the literature needs to be more consistent on the appropriateness of patellar resurfacing during TKA. Although there is no evidence that resurfacing has a clinically significant difference in patient-reported outcome measures (PROMs), it is a cost-effective procedure that reduces re-intervention rates and is associated with few complications [16, 17]. Implant design has a key role. Single central pin and cementless metal-supported patellar prosthesis are considered important stress factors; for these reasons, a three-pin patellar component with an all-polyethylene dome implanted with cement was introduced [3, 4, 8, 9]. Finally, more constrained implants are characterized by a PPPF higher risk [4, 9, 14].

#### Surgery-related risk factors

Surgery-related factors include the phases from the patella exposure to the prosthetic knee implantation. One of the main issues analyzed in the literature is patella devascularization which may lead to osteonecrosis and increased fracture risk [3, 4, 8, 9, 18, 19]. Devascularization could result from several surgical procedures, particularly medial parapatellar arthrotomy (MPA), infrapatellar fat pad excision, and lateral release [3, 4, 8, 9, 18].

The patella blood supply is derived from an extraosseous peripatellar anastomotic ring from which intraosseous vessels flow [18, 19]. Lazaro et al. [18] demonstrated that MPA completely disrupts medial patellar blood flow leaving vascularization to the lateral vessels only; the authors additionally described the presence of the supreme geniculate artery within the vastus medialis oblique (VMO) discouraging a midvastus approach and suggested a subvastus approach. Moreover, leaving at least one centimeter from the patellar margin could result in less compromised blood flow [18]. Excessive fat pad excision may contribute to patella devascularization because its removal interrupts the inferior polar vessels and the transverse infrapatellar branch. For this reason, the authors suggest removing only the necessary fat pad for obtaining good tibial exposure [18]. Finally, Lazaro et al. proposed preserving the peripheral border of the lateral meniscus not to damage the inferolateral geniculate artery [18].

The lateral release is also associated with devascularization [3, 4, 18]. Chalidis et al. [4] reported 51.2% of PPPF related to this procedure. Lazaro et al. [18] described a sacrifice of lateral geniculate arteries when performing extended-release. Therefore, the authors proposed to leave a lateral border around the patella of at least 15 mm and to complete the release distal to the superolateral border of the patella and proximal to the joint line. To preserve the prepatellar vessels, all tissue flaps created during the surgical procedure should be superficial to the anterior patellar periosteum [18].

Conversely, Hempfing et al. [20] did not report a significant patellar blood flow reduction due to soft tissue dissection, but the authors described a correlation between 100° knee flexion, patellar eversion, and decreased blood flow. The same results were described by Stoffel et al. [21] in their paper. Technically, eversion is used to improve exposure. Stoffel et al. measured, through Doppler ultrasound, 87% ischemia grade during eversion compared with 47% in a slight retraction; therefore, the authors recommended only slight retraction for patella exposure [21]. Many authors considered thermal necrosis associated with cement polymerization another important risk factor for PPPF [4, 8, 9, 14].

Patella thickness is another important risk factor. Surgeons should restore the native thickness, avoiding over-sectioning, rising fracture risk, and under-sectioning, leading to overload with flexion loss and increased joint reaction forces [3, 4, 8, 9, 22, 23]. Reuben et al. [24] described a residual patellar thickness of at least 15 mm as the minimum bone reserve to avoid overstressing and fracture risk.

A correct TKA implant positioning and extensor mechanism alignment are essential to avoid increased joint reaction forces and the PPPFs risk; a flexed femoral component or large anteroposterior diameter predisposes to PPPFs [3, 8, 9, 14, 25]. Seo et al. reported an increased fracture risk in significant preoperative mechanical malalignment, shorter postoperative patellar tendon length, and lower postoperative Insall-Salvati ratio [26]. Finally, the literature agrees that rTKA is a well-known independent PPF risk factor [3–5].

### Clinical manifestation and diagnosis

PPPFs may be asymptomatic at clinical presentation and be discovered on radiographs during outpatient follow-up [1, 4, 8–10]. Chalidis et al. [4] reported that more than 80% of PPPFs are diagnosed when symptoms occur, mainly consisting of anterior knee pain associated with swelling and patellar tenderness [9, 14]. Patients may complain of other manifestations, such as knee instability with extensor system weakness [9]. Diagnosis is usually made by anteroposterior and lateral X-ray view and Merchant X-ray view [9, 12, 14]. A CT scan may be performed to evaluate better any implant loosening and fracture morphology [12, 14]. Technetium-99m (Tc-99m) bone scan could be performed to discover occult fractures or to distinguish an old fracture from a recent one [9, 14]. Finally, the patient’s history, blood tests, and comparison with previous radiographs are critical in directing the surgeon to aseptic loosening, infection-related loosening, or upcoming implant failure [12].

### Classification systems

Several classification systems for PPPFs are described in the literature, but the most widely used are those of Goldberg et al. [6] and Ortiguera and Berry [1].

**Fig. 3:**
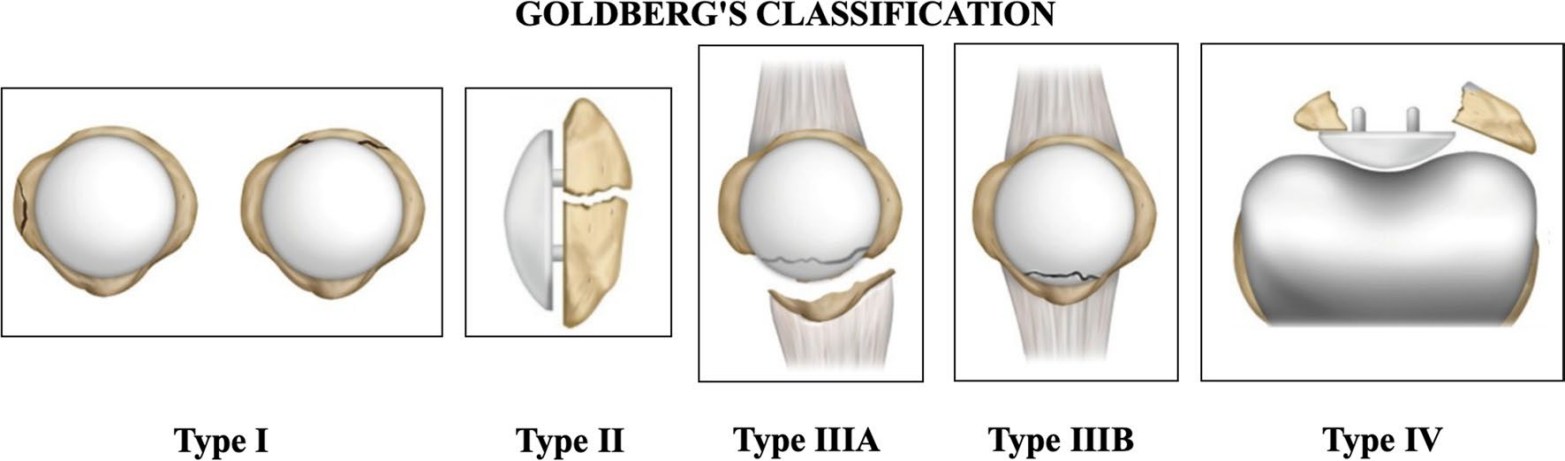
Goldberg’s classification

Goldberg’s classification categorizes fractures according to fracture pattern, extensor mechanism integrity, and patellar component stability (Fig. 3). Type I is characterized by patellar fractures with intact extensor apparatus and implant stability, while in type II, one or both previously mentioned elements are damaged. Type III is divided into IIIa, in which the fracture of the inferior pole of the patella is associated with a patellar tendon injury, and IIIb, in which the same fracture pattern has an intact patellar tendon. Type IV consists of patellar fracture-dislocations [6].

**Fig. 4:**
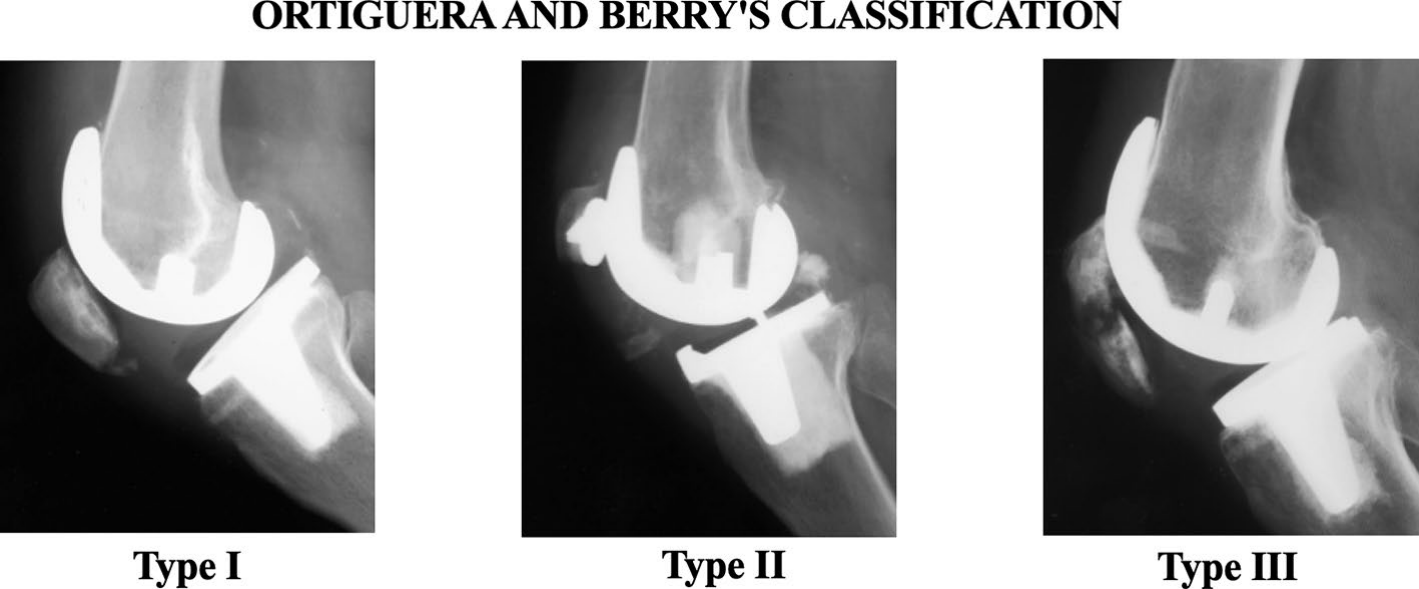
Ortiguera and Berry’s classification

Ortiguera and Berry’s classification is the most recent (Fig. 4) (Table 2); it considers the amount of residual patellar bone, implant stability, and extensor apparatus integrity. Type I fractures have a stable implant and an intact extensor apparatus; type II disrupts the extensor mechanism; type III has a mobilized implant but an intact extensor apparatus. The latter group is further divided into IIIa, characterized by a good amount of residual bone reserve for revision, and IIIb, with poor bone reserve, defined as a bone thickness of < 10 mm or marked comminution of the patellar component. If disruption of the extensor mechanism and a loosened component are concomitant, it is classified as type II [1].

**Table 2:** Ortiguera and Berry periprosthetic patellar fractures classification

I	Stable implant and intact extensor mechanism
II	Disruption of the extensor mechanism with or without implant loosening
III	Loose implant with integrity of the extensor apparatus (a) Good bone stock (b) Poor bone stock (bone thickness of < 10 mm or marked comminution)

Considering atraumatic fatigue fractures, Windsor et al. [10] associated the fracture pattern with the causative mechanism. Horizontal fractures were related to patellar maltracking and dislocation, while vertical ones passed through the patellar fixation holes. Comminuted and dislocated fractures were usually a whole of transverse and vertical fractures [10].

### Outcomes and treatment strategies

Conservative treatment consists mainly of immobilization in extension, with a cast or brace, for four to six weeks, depending on the fracture type; full or partial weight-bearing is allowed [1–4, 6–10, 14]. Surgical procedures may include open reduction and internal fixation (ORIF), partial or complete patellectomy with the extensor apparatus repair, patellar component revision, or extensor apparatus reconstruction [1–9, 14, 27–29].

**Fig. 5:**
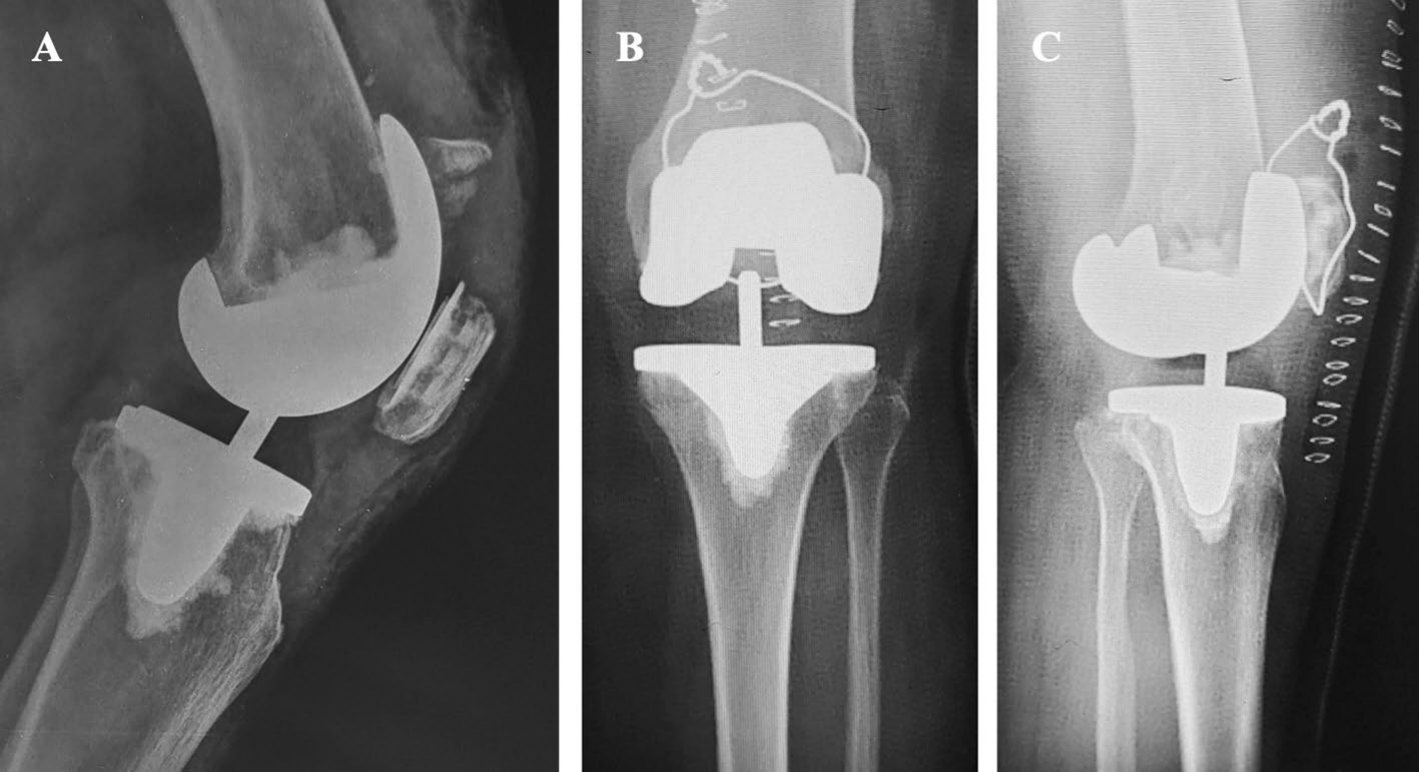
Periprosthetic patellar fracture in the resurfaced setting with failure of the extensor apparatus. **A** Preoperative lateral X-ray shows a PPPF with extensor mechanism rupture and rotating hinge knee system (Depuy) with stable patellar implant; **B** Postoperative anteroposterior X-ray view shows the fracture is reduced and fixed with cerclage wiring; **C** Lateral X-ray view shows good approximation

**Fig. 6:**
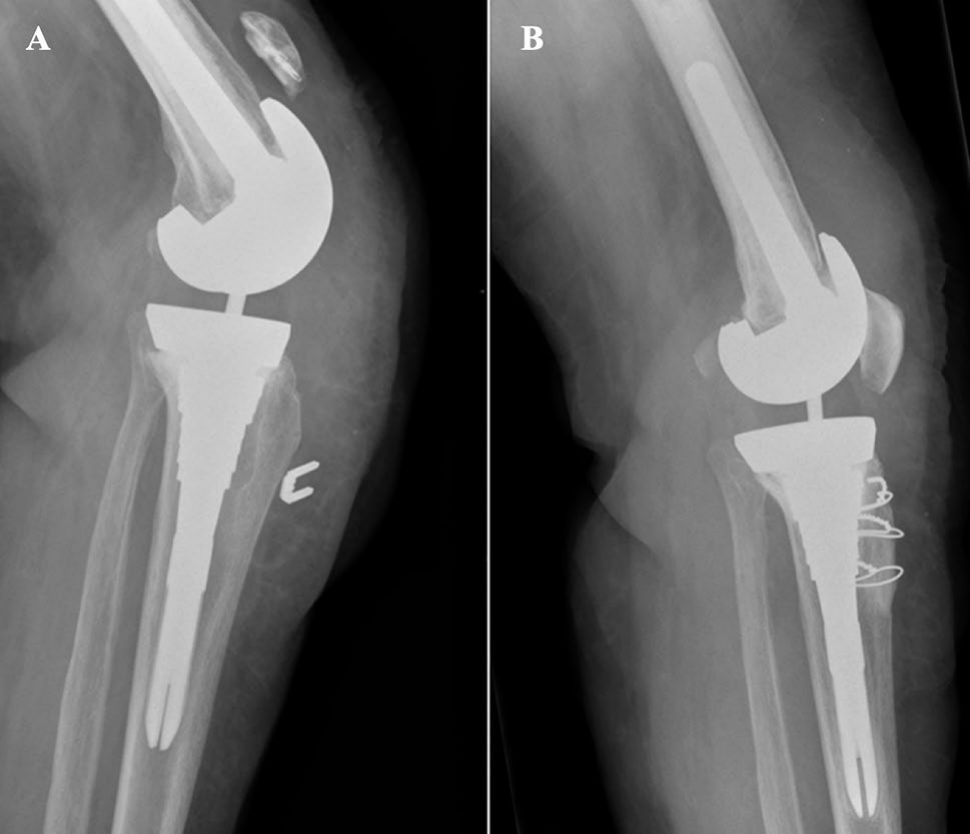
Periprosthetic patellar fracture in the resurfaced setting with failure of the extensor apparatus and reconstruction using allograft. **A** Preoperative lateral X-ray shows the periprosthetic patellar fracture in the resurfaced setting with failure of the extensor apparatus; **B** Postoperative lateral X-ray shows reconstruction using extensor mechanism allograft

Although several algorithms were developed, in the literature, there is a general trend in favor of nonsurgical treatment, except in cases of implant loosening or extensor apparatus failure (Table 3) [1, 4]. In Chalidis et al. [4] systematic review, 68.8% of PPPFs received non-operative treatment, and only 31.2% of patients underwent surgery. Conservative management is favored in most cases because of the higher number of complications associated with surgery, such as the infection rate reported in 19.2% of revision surgeries [4]. Conservative treatment is characterized by good functional results without pain, patellar, or extensor instability, with few cases of minimal extensor delay of about 5° [1–4, 9, 14]. Although knee function was restored, non-union or fibrous union was detected in many cases on radiographic follow-up [1, 8]. Conversely, surgery is required in type II and III PPPFs, according to Ortiguera and Berry’s classification, characterized by extensor mechanism failure or implant loosening (Fig. 5) [1]. In type II fractures, surgical options are patellar fragments ORIF, partial or complete patellectomy with extensor apparatus repair, augmentation with adjacent tendons, or reconstruction with extensor allograft in cases of severe extensor tendon rupture and residual extension lag (Fig. 6) [1–4, 8, 9, 14, 15]. Type III fractures involving implant loosening require operative treatment, especially in symptomatic patients [1–4, 8, 9]. The most appropriate management is related to residual bone stock: type IIIa fractures undergo implant revision or resection arthroplasty, while type IIIb requires implant removal with partial or complete patellectomy [1, 4, 28, 29]. However, the complication rate is high; Ortiguera and Berry [1] described more than half of type III fractures as symptomatic at the last follow-up, while Parvizi et al. [3] reported that two out of three type III PPPFs required reoperation. Sometimes, in low functional demands and non-symptomatic patients, even PPPF type II and III may be treated conservatively due to the high complication rate associated with surgical procedures [1, 4].

**Table 3:** Algorithm for the proper management of periprosthetic patellar fractures

Fracture pattern	Management options
Fracture: undisplaced	• Cast
Implant: stable	• Functional brace
Fracture: displaced	• Partial patellectomy
Implant: stable	• Non-operative management
Fracture: ± displaced	• Available bone stock
Implant: loose	• Revision + patelloplasty
	• Excision arthroplasty
	• Non-available bone stock
	• Excision arthroplasty
	• Partial or total patellectomy

Intraoperative PPPFs and fractures occurring in non-resurfaced patella deserve a separate analysis. Intraoperative PPPFs could be synthesized during surgery, and the patella should be later resurfaced if symptoms appear [11], whereas non-resurfaced PPPFs are generally treated as traumatic patellar fractures [3, 8, 30].

Windsor et al. [10] in their study, treated PPPFs surgically or conservatively based on fracture patterns and displacement. The authors divided fractures into the transverse, vertical, and comminuted, and further into displaced or nondisplaced with 2 cm as the displacement limit. Vertical, comminuted, and transverse fractures with less than 2 cm displacement could be treated conservatively; on the contrary, both transverse patella fractures with displacement greater than 2 cm and severely comminuted fractures associated with extension lag and quadriceps weakness should be managed surgically [10].

## Conclusions

The PPPF is an uncommon but potentially devastating complication after TKA and rTKA. Risk factors are well established, and surgeons should be aware of them to prevent this unpleasant complication. Most PPPFs are asymptomatic; therefore, there is a general trend in favor of nonsurgical treatment, which often gives satisfactory clinical and functional results, except in cases of implant loosening or disruption of the extensor mechanism require surgery for proper restoration of normal extensor function and implant stability.

## Data Availability

The dataset analyzed in this study is available from the corresponding author on reasonable request.

## Abbreviations

PPPFs: Periprosthetic patellar fractures
TKA: Total knee arthroplasty
TKR: Total knee replacement
rTKA: Revision total knee arthroplasty
RA: Rheumatoid arthritis
mm: Millimeters
cm: Centimeters
MA: Medial arthrotomy
VMO: Vastus medialis oblique
CT: Computed tomography
Tc99m: Technetium-99-metastable
ROM: Range of motion
ORIF: Open reduction and internal fixation
PROMs: Patient-reported outcome measures
PS: Posterior-stabilized
CR: Cruciate retaining
PMMA: Poly-methyl-methacrylate
AP: Anteroposterior
